# Supplementary material for: The paraspeckle protein NONO potentiates the antiviral innate immune response through chromatin regulation
Source: bioRxiv. 2026 Jul 14:2026.07.11.737985. Preprint. [Version 1] doi: 10.64898/2026.07.11.737985 (PMC13405385; doi:10.64898/2026.07.11.737985)

bioRxiv preprint doi: https://doi.org/10.64898/2026.07.11.737985; this version posted July 14, 2026. The copyright holder for this preprint (which was not certified by peer review) is the author/funder. This article is a US Government work. It is not subject to copyright under 17 USC 105 and is also made available for use under a CC0 license.

Figure S1. NONO loss enhances orthoflavivirus replication.
(A-B) CRISPR knockout efficiency of (A) NONO protein (immunoblot) and (B) *NONO* mRNA (qRT-PCR). (C) Infectious viral titers from WT or *NONO* KO A549 cells infected with yellow fever virus (YFV) Asibi MOI=0.001, Kyasanur Forest disease virus (KFDV) MOI=0.01, and Dengue virus serotype 2 (DENV-2) NGC MOI=1 at 48 hpi (plaque assay). (D-E) *ZIKV* and *YFV-17D* expression from infected WT or *NONO* KO A549 cells from (A) (qRT-PCR). (F-G) Levels of ZIKV-NS5 and YFV-17D-NS3 protein from infected WT or *NONO* KO A549 cells from (A) (immunoblot). (H-L) WT or *NONO* KO A549 cells infected with WNV-NY99 MOI=0.0001. (H) *IFN-β* and *IL-6* expression (qRT-PCR). (I) IFN-β protein in supernatants (ELISA). (J) *IFI6* and *RSAD2* expression (qRT-PCR). (K) *WNV* expression (qRT-PCR). (L) Levels of WNV-NS5 protein (immunoblot). Data are expressed as means (n=3) ± SD, *p < 0.05, **p < 0.01, ***p < 0.001, ****p < 0.0001 (Student's t-test or two-way ANOVA with Sidak's multiple comparisons) and are representative of 2-3 independent experiments.

Figure S2. NONO is critically required for establishing an effective antiviral response.
Infectious viral titers from WT or *NONO* KO A549 cells pretreated with Ruxolitinib (10 μg/mL) or DMSO carrier for 1 hour before infection with Zika virus (ZIKV) Dakar MOI=0.01 (plaque assay). Data are expressed as means (n=3) ± SD, *p < 0.05, **p < 0.01, ***p < 0.001, ****p < 0.0001 (two-way ANOVA with Tukey's multiple comparisons) and are representative of 2-3 independent experiments.

Figure S3. NONO broadly promotes innate immune responses.
(A-C) WT or *NONO* KO A549 cells infected with SeV (100 HAU/mL) for 8 hours. (A) Reads Per Kilobase Million (RPKM) for *IFN-β*. (B-C) GSEA terms and pathways of upregulated transcripts from SeV-infected (B) and mock (C) samples. (D-E) siRNA silencing efficiency of (D) NONO protein (immunoblot) and (E) *NONO* mRNA (qRT-PCR). (F) *IFN-β*, *IL-6*, and *IFI6* expression from WT or *NONO* KO A549 cells stimulated with Poly(dA:dT) (1 μg/mL) (qRT-PCR). (G) A graphical summary using Ingenuity Pathway Analysis (IPA) performed by importing the differential gene expression list comparing Mock to SeV-infected WT cells visualizes the most significantly enriched pathways, upstream regulators, and biological functions, without the addition of external nodes. Data are expressed as means (n=3) ± SD, *p < 0.05, **p < 0.01, ****p < 0.0001 (Student's t-test or two-way ANOVA with Sidak's multiple comparisons) and are representative of 2-3 independent experiments.

Figure S4. NONO augments innate immunity specifically at the transcriptional level.
(A) *NONO* expression from WT or *NONO* KO A549 cells infected with SeV (100 HAU/mL). (B) *NONO* expression from WT or *NONO* KO A549 cells stimulated with IFN-β (100 IU/mL). (C) Subcellular fractionation of NONO and WNV-NS5 protein from WT A549 cells infected with WNV-Bird 114 MOI=0.0001 (immunoblot).

bioRxiv preprint doi: https://doi.org/10.64898/2026.07.11.737985; this version posted July 14, 2026. The copyright holder for this preprint (which was not certified by peer review) is the author/funder. This article is a US Government work. It is not subject to copyright under 17 USC 105 and is also made available for use under a CC0 license.

(D) Endogenous co-immunoprecipitation of NONO from WT A549 cells stimulated with IFN-β (1000 IU/mL) for 8 hours. (E) rMATS alternative splicing events between WT and *NONO* KO A549 cells. (F-G) Mature and pre-mRNA expression for *MX1* from WT or *NONO* KO A549 cells stimulated with IFN-β (100 IU/mL) for 8 hours (qRT-PCR). (H-J) Sashimi plots for *MX1*, *RSAD2*, and *IL-6* from WT or *NONO* KO A549 cells mock or SeV infected (100 HAU/mL) for 8 hours. (K) rMATS top 10 differentially spliced genes from WT and *NONO* KO A549 cells. Data are expressed as means (n=3) ± SD, **p < 0.01, ****p < 0.0001 (two-way ANOVA with Sidak's multiple comparisons or Student's t-test) and are representative of 2-3 independent experiments.

Figure S5. NONO enhances chromatin accessibility of innate immune genes.
(A-C) ATAC-seq of WT or *NONO* KO A549 cells infected with SeV (100 HAU/mL) for 8 hours (n=4 per group). (A-B) Genome browser view of open chromatin peaks for *IL-6*, *IL6-AS1*, *RSAD2*, and *CMPK2* with highlighted portions indicating regions with significantly less available chromatin in the SeV-infected *NONO* KO compared to the SeV-infected WT. (C) HOMER *de novo* motif analysis for depleted motifs in mock *NONO* KO compared to WT samples.

Figure S6. NONO loss impairs RNA Pol II binding to innate immune gene promoters.
(A-B) WT or *NONO* KO A549 cells infected with SeV (100 HAU/mL) for 8 hours. (A) ChIP-qPCR showing H3K27me3 and H3K4me3 histone modifications at *RPL30* and *α-Satellite*. (B) ChIP-qPCR showing RNA-Pol II occupancy at *RPL30* and *α-Satellite*. (C) ChIP-qPCR showing RNA-Pol II occupancy at *RPL30* and *α-Satellite* for WT or *NONO* KO A549 cells stimulated with IFN-β (1000 IU/mL) for 8 hours. Data are expressed as means (n=3) ± SD and are representative of 2-3 independent experiments.

bioRxiv preprint doi: https://doi.org/10.64898/2026.07.11.737985; this version posted July 14, 2026. The copyright holder for this preprint (which was not certified by peer review) is the author/funder. This article is a US Government work. It is not subject to copyright under 17 USC 105 and is also made available for use under a CC0 license.

Table S1: Primer sequences

REAGENT or RESOURCE	FORWARD	REVERSE
Oligonucleotides for qRT-PCR		
18S rRNA	5’-GTAACCCGTTGAACCCCATT-3’	5’-CCATCCAATCGGTAGTAGCG-3’
IFNB1	5’-TCTGGCACAACAGGTAGTAGGC-3’	5’-GAGAAGCACAACAGGAGAGCAA-3’
IL-6	5’-AGACAGCCACTCACCTCTTCAG-3’	5’-TTCTGCCAGTGCCTCTTTGCTG-3’
IFI6	5’-TGATGAGCTGGTCTGCGATCCT-3’	5’-GTAGCCCATCAGGGCACCAATA-3’
RSAD2	5’-CCAGTGCAACTACAAATGCGGC-3’	5’-CGGTCTTGAAGAAATGGCTCTCC-3’
IFIT1	5’-GCCTTGCTGAAGTGTGGAGGAA-3’	5’-ATCCAGGCGATAGGCAGAGATC-3’
IFIT2 [63]	5’-ATGTGCAACCTACTGGCCTAT-3’	5’-TGAGAGTCGGCCCATGTGATA-3’
NONO	5’-CATCAAGGAGGCTCGTGAGAAG-3’	5’-TGGTTGTGCAGCTCTTCCATCC-3’
OAS1	5’-AGGAAAGGTGCTTCCGAGGTAG-3’	5’-GGACTGAGGAAGACAACCAGGT-3’
MX1	5’-GGCTGTTTACCAGACTCCGACA-3’	5’-CACAAAGCCTGGCAGCTCTCTA-3’
IFITM1 [64]	5’-TGACCATTGGATTCATCCTG-3’	5’-TGCACAGTGGAGTGCAAAG-3’
WNV [65]	5’-TCAGCGATCTCTCCACCAAAG-3’	5’-GGGTCAGCACGTTTGTCATTG-3’
ZIKV	5’-CTGTGGCATGAACCCAATAG-3’	5’-ATCCCATAGAGCACCACTCC-3’
YFV	5’-CAATGCTCTTGGACATGGAGG-3’	5’-CTGAAGACTTTCCTCTGGTCATCCC-3’
IFI6 pre-mRNA [66]	5’-TGATCCCTGGCTTGTGAACC-3’	5’-GATGTCTGCCATGGAAACGC-3’
MX1 pre-mRNA [67]	5’-ACTACGACCGCAGAGCTGAACCTT-3’	5’-AGGCCCCACTCCAGACCCACA-3’
Oligonucleotides for ChIP-PCR		
IFNB1 Promoter [68]	5’-GAAAGGGAGAAGTGAAAGTGG-3’	5’-AAGGCTTCGAAAGGTTGCAG-3’

bioRxiv preprint doi: https://doi.org/10.64898/2026.07.11.737985; this version posted July 14, 2026. The copyright holder for this preprint (which was not certified by peer review) is the author/funder. This article is a US Government work. It is not subject to copyright under 17 USC 105 and is also made available for use under a CC0 license.

IFIT1 5'-UTR [69]	5'-CTTTCCCCTTTCGGTTTCCCTA-3'	5'-TGGCTATTCTGTCTTGTGGATTTTCA-3'
DDX58 5'-UTR [69]	5'-CATTTCACACCGACCAAGATGGAA-3'	5'-GCCACCTGTTGTCCAAAATGACTG-3'
MX1 Promoter	Cell Signaling Technology Cat# 57949	
RPL30	Cell Signaling Technology Cat# 7014	
α-Satellite	Cell Signaling Technology Cat# 4486	

bioRxiv preprint doi: https://doi.org/10.64898/2026.07.11.737985; this version posted July 14, 2026. The copyright holder for this preprint (which was not certified by peer review) is the author/funder. This article is a US Government work. It is not subject to copyright under 17 USC 105 and is also made available for use under a CC0 license.

FIG S1

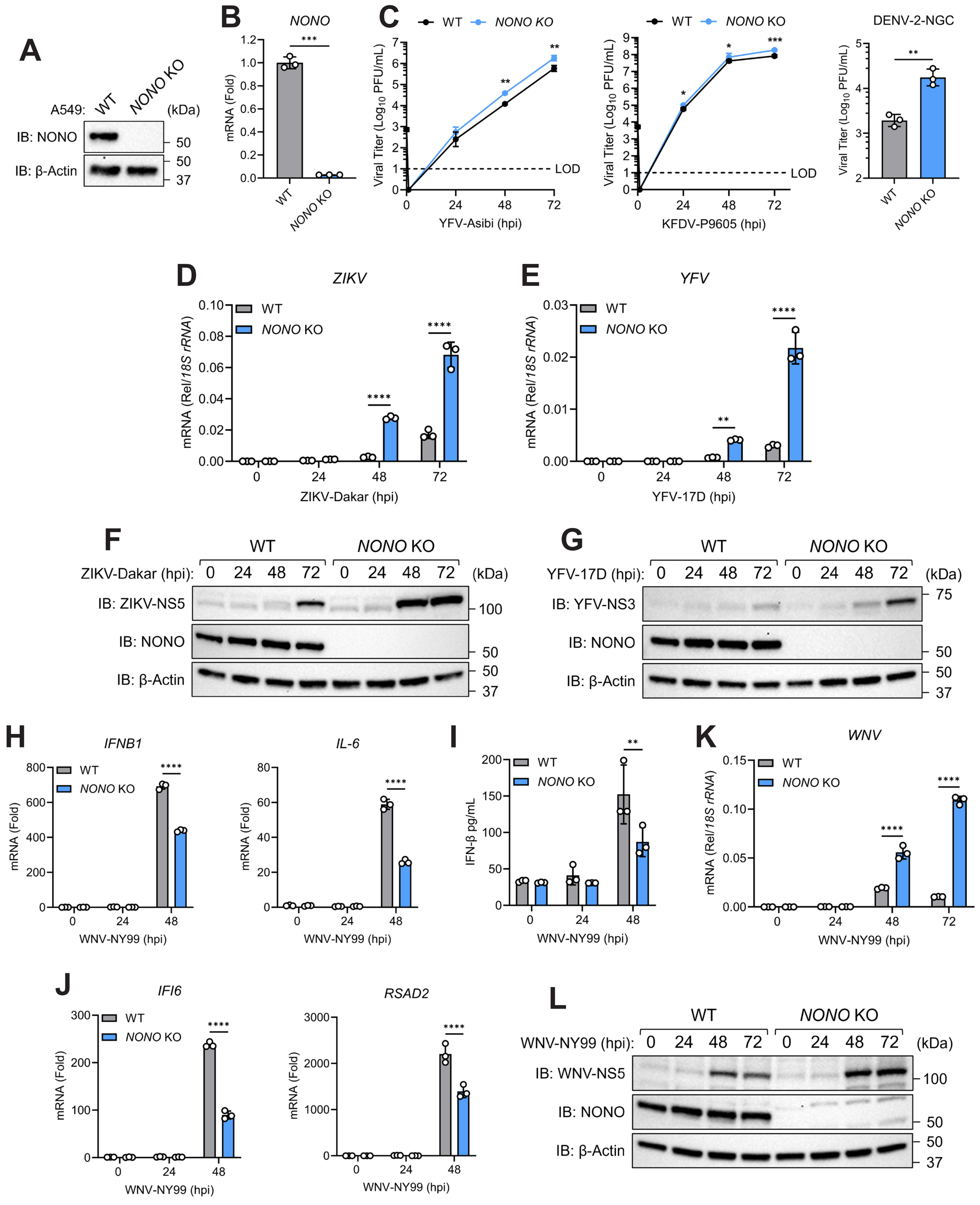

bioRxiv preprint doi: https://doi.org/10.64898/2026.07.11.737985; this version posted July 14, 2026. The copyright holder for this preprint (which was not certified by peer review) is the author/funder. This article is a US Government work. It is not subject to copyright under 17 USC 105 and is also made available for use under a CC0 license.

FIG S2

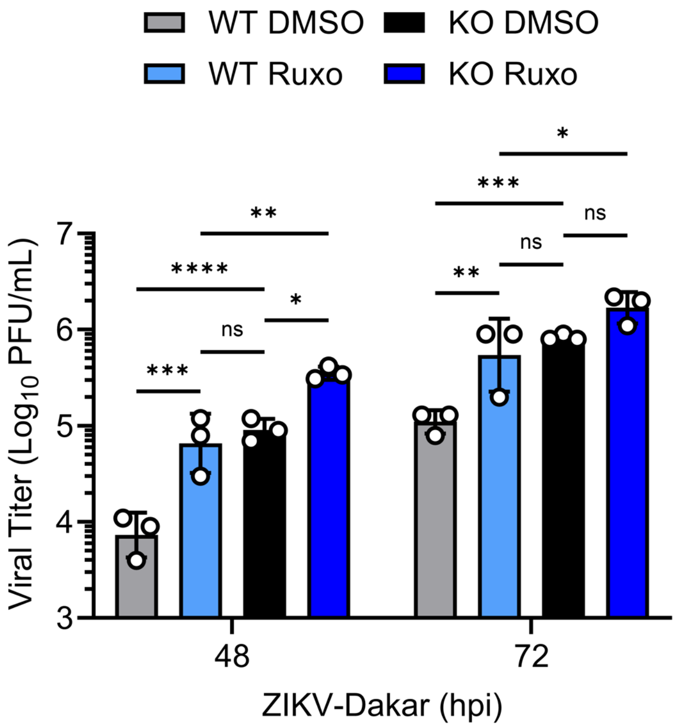

bioRxiv preprint doi: https://doi.org/10.64898/2026.07.11.737985; this version posted July 14, 2026. The copyright holder for this preprint (which was not certified by peer review) is the author/funder. This article is a US Government work. It is not subject to copyright under 17 USC 105 and is also made available for use under a CC0 license.

FIG S3

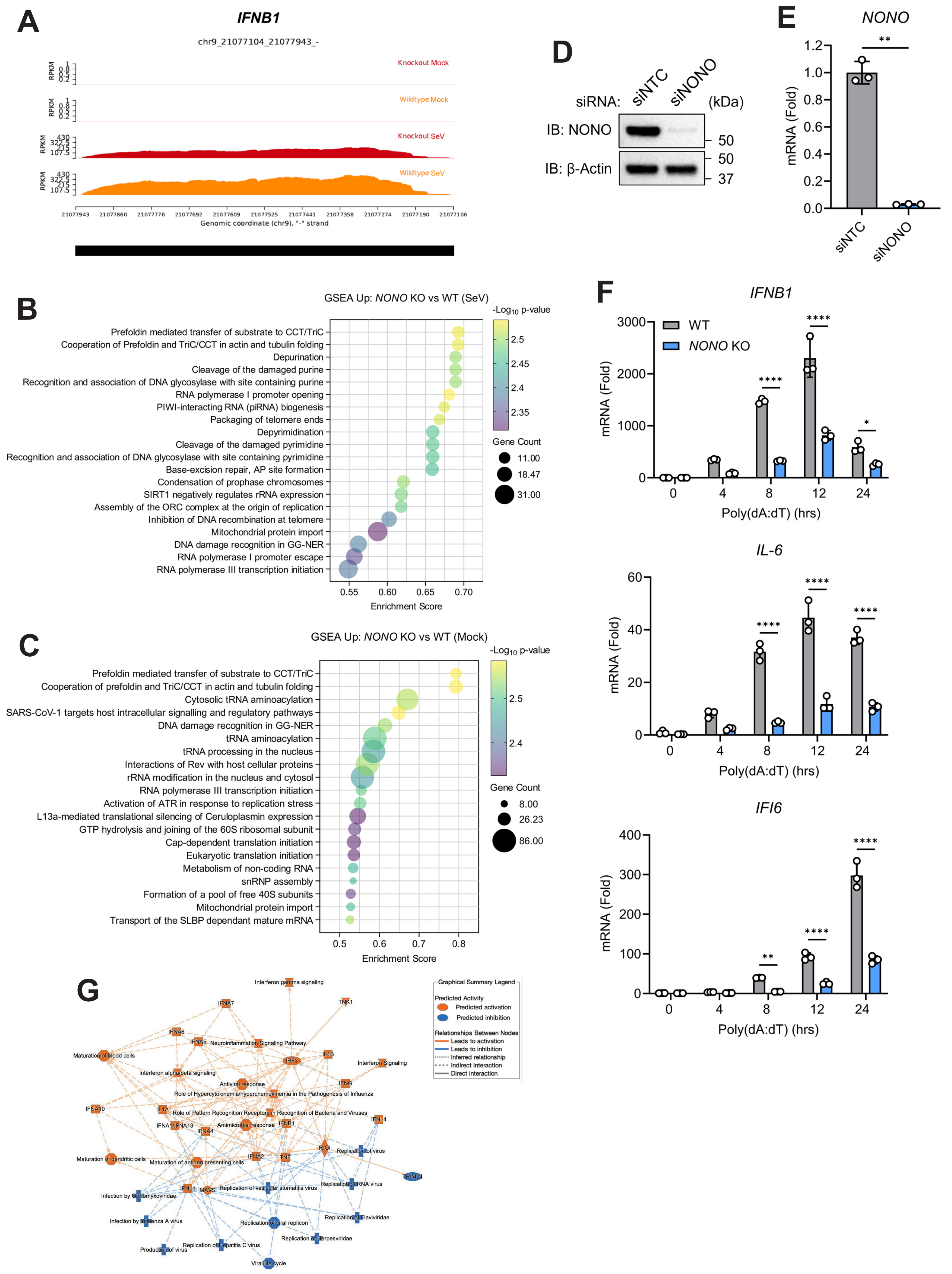

bioRxiv preprint doi: https://doi.org/10.64898/2026.07.11.737985; this version posted July 14, 2026. The copyright holder for this preprint (which was not certified by peer review) is the author/funder. This article is a US Government work. It is not subject to copyright under 17 USC 105 and is also made available for use under a CC0 license.

FIG S4

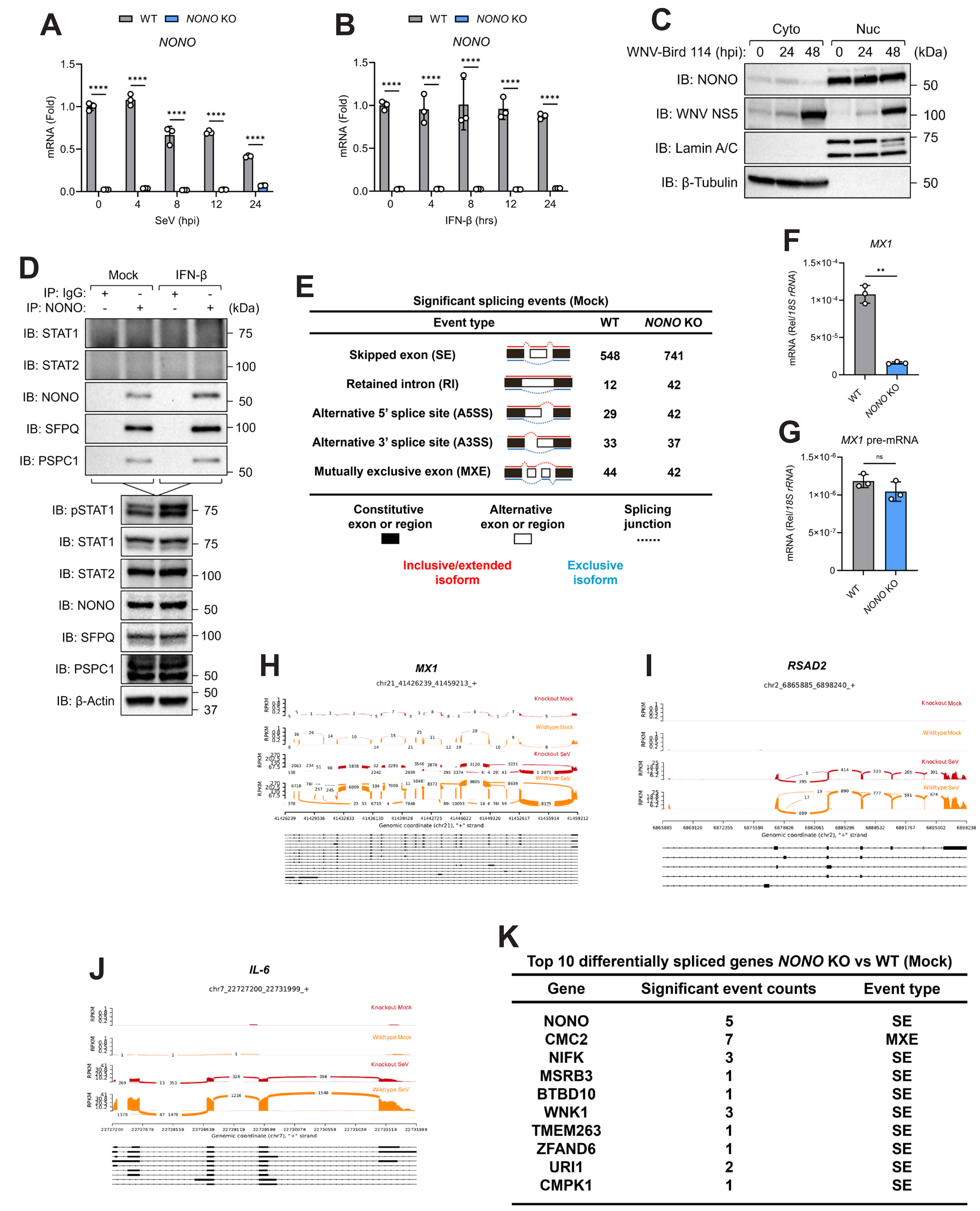

Significant splicing events (Mock)

Event type	WT	NONO KO
Skipped exon (SE)	548	741
Retained intron (RI)	12	42
Alternative 5' splice site (A5SS)	29	42
Alternative 3' splice site (A3SS)	33	37
Mutually exclusive exon (MXE)	44	42

Constitutive exon or region — Alternative exon or region — Splicing junction

Inclusive/extended isoform — Exclusive isoform

Top 10 differentially spliced genes *NONO* KO vs WT (Mock)

Gene	Significant event counts	Event type
NONO	5	SE
CMC2	7	MXE
NIFK	3	SE
MSRB3	1	SE
BTBD10	1	SE
WNK1	3	SE
TMEM263	1	SE
ZFAND6	1	SE
URI1	2	SE
CMPK1	1	SE

bioRxiv preprint doi: https://doi.org/10.64898/2026.07.11.737985; this version posted July 14, 2026. The copyright holder for this preprint (which was not certified by peer review) is the author/funder. This article is a US Government work. It is not subject to copyright under 17 USC 105 and is also made available for use under a CC0 license.

FIG S5

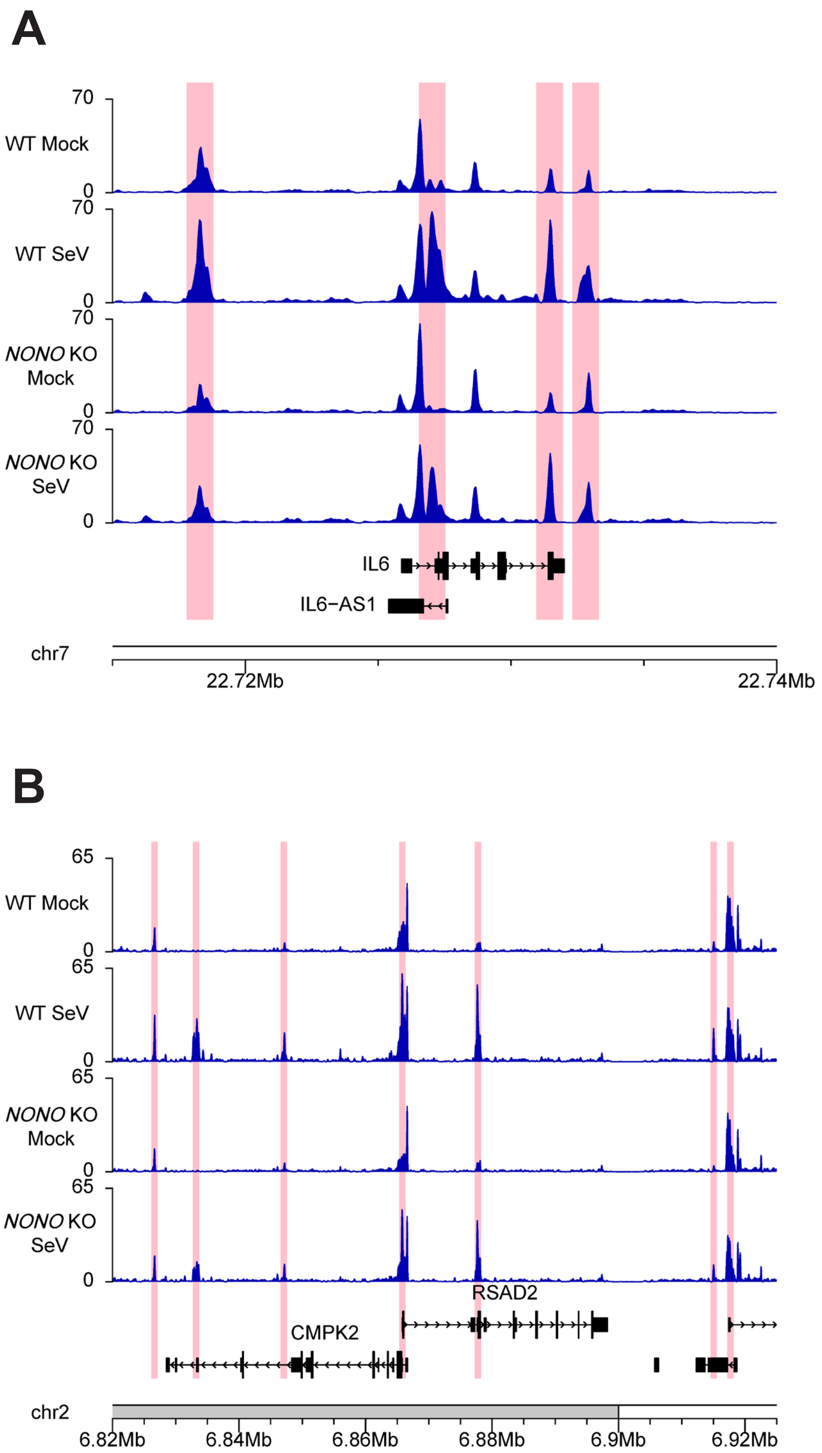

C

Depleted in *NONO* KO vs WT (Mock)

HOMER *de novo* Motif	Name	P-value	% of Targets	% of Background	Rank
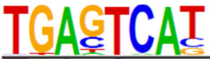	FOS	1e-1988	61.32%	11.66%	#1
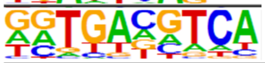	ATF2	1e-61	19.65%	12.40%	#4

bioRxiv preprint doi: https://doi.org/10.64898/2026.07.11.737985; this version posted July 14, 2026. The copyright holder for this preprint (which was not certified by peer review) is the author/funder. This article is a US Government work. It is not subject to copyright under 17 USC 105 and is also made available for use under a CC0 license.

FIG S6

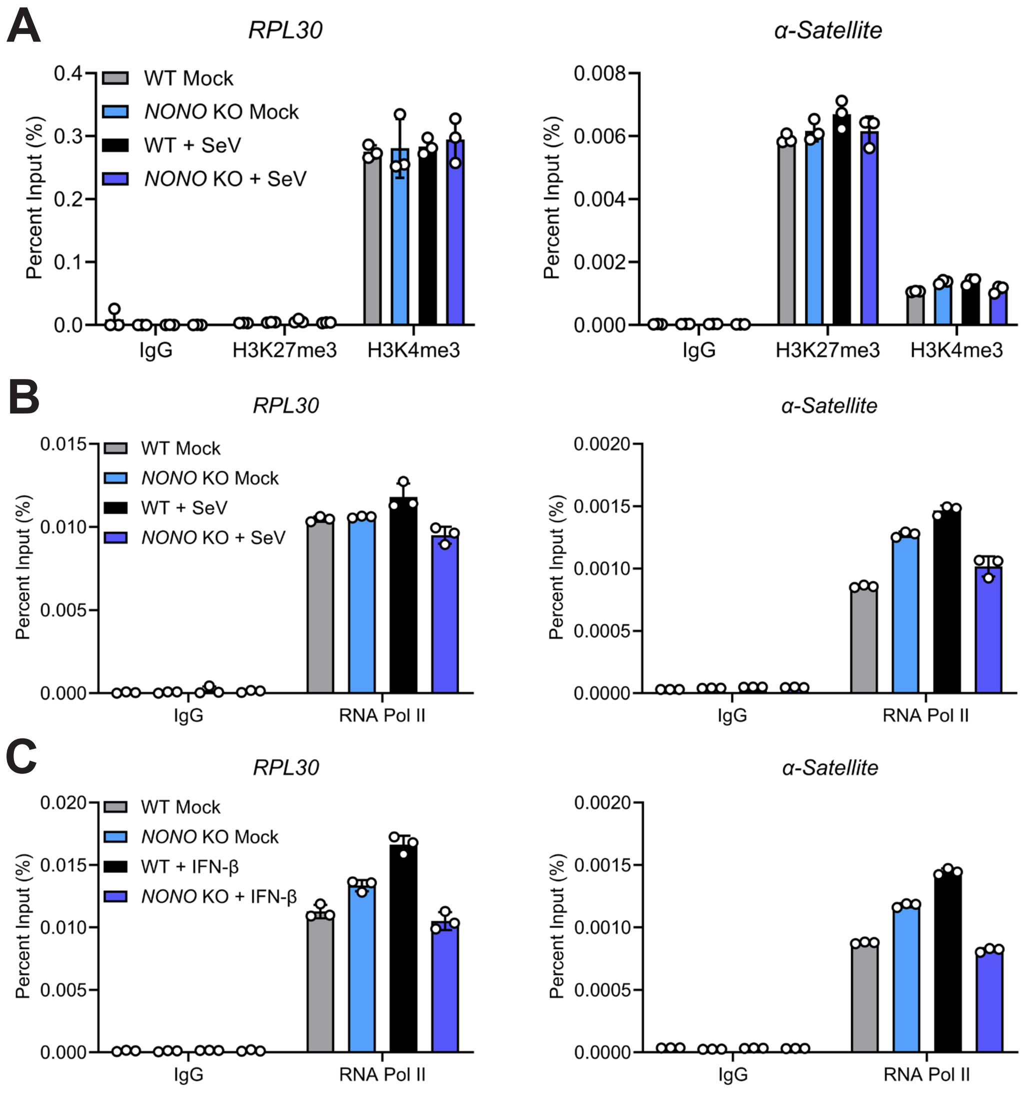

Supplement: Supplement 1 [file NIHPP2026.07.11.737985v1-supplement-1.pdf]
